# Solution-blown PAN/Congo red nanofibrous sensors for colorimetric detection of soybean oil oxidation

**DOI:** 10.1038/s41538-026-01157-z

**Published:** 2026-10-05

**Authors:** Shamous M. Ibrahim, Ayat F. Hashim, Ayman El Said, Tamer Hamouda

**Affiliations:** 1 Textile Department, Faculty of Applied Arts, Capital University (formerly Helwan University), Giza, Egypt; 2 Fats and Oils Department, National Research Centre, Giza, Egypt; 3 Textile Research and Technology Institute, National Research Centre, Giza, Egypt

**Keywords:** Chemistry, Engineering, Environmental sciences, Materials science, Nanoscience and technology

## Abstract

Recognizing the importance of cooking oil quality and the potential health risks associated with oxidation, this study developed an indirect-contact nanofiber-based system for detecting cooking oil oxidation. The effects of spinning parameters on sensor sensitivity were investigated. Eight nanofiber mats (MAT I–VIII) were fabricated using different needle sizes (21 G and 18 G), polymer concentrations (12% and 15%), and air pressures (0.5 and 2 MPa). Congo red was incorporated into polyacrylonitrile (PAN) nanofiber mats using a solution-blowing technique. The mats were characterized by scanning electron microscopy, Fourier transform infrared spectroscopy, X-ray diffraction, and colorimetric analysis. Sensor performance was evaluated during soybean oil heating at 180 ± 2 °C for 7 h. Peroxide value, para-anisidine value, total oxidation value, and acid value were monitored as oxidation indicators. After 7 h, peroxide value, para-anisidine value, and acid value increased to 14.55 meq O_2_/kg oil, 9.48, and 0.84 mg KOH/g, respectively. Nanofiber diameter strongly influenced oxidation detection. MATs VI–VIII, produced with the smaller 21 G needle, exhibited faster and more stable responses. MAT VIII (21 G, 15% PAN, 0.5 MPa) showed the most favorable sensitivity and response stability. The findings demonstrate the potential of PAN/Congo red nanofiber mats as an indirect-contact approach for visual monitoring of cooking oil oxidation.

## Introduction

Over the past few years, interest in the production of nanofibers has grown due to their diverse industrial applications^1^. As a result of the unique properties of nanofibers, the scope of research and development in their application areas has expanded. The diameter of nanofibers is influenced by several factors, including the type of polymer used and the fiber production method. Nanofibers are useful in a variety of applications due to their unique properties, such as light weight and high surface area-to-volume ratio. Tissue engineering, protective clothing, energy storage, filtration, sensors, and functional materials are types of applications that can use nanofibers^2^.

There are several techniques for producing nanofibers, including Electro-Spinning (ES), Solution-Blown Spinning (SBS), centrifugal spinning, and melt-blown spinning. Electrospinning is the most widely used nanofiber production technology because it produces fibers with high specific surface areas and nano diameters. On the other hand, this technology is hampered by its need for a high-voltage electric field, in addition to limitations on its production and deposition method when rapid manufacturing is required. SBS has emerged as a rapid nanofiber production alternative to electrospinning, where the polymer solution is expanded by a high-speed gas stream instead of an electric field. Solution blowing is an economical and inexpensive technique for producing nanofibers. Nanofibers produced using this technique are widely used in many applications. The solution blowing technique is characterized by high production rates and low labor requirements. These characteristics make it suitable for manufacturing flexible and porous nanofiber mats for practical sensing applications and food packaging^3^.

Solution blowing technology was utilized to produce nanomaterials for food-related applications, particularly active and smart food packaging. Natamycin-containing nanomaterials were produced for use in active packaging as an antimicrobial agent^4^. Smart nanofiber mats incorporating anthocyanin-rich extracts derived from agricultural food waste were developed and exhibited a response to pH and ammonia vapor via visible color changes. This highlights the potential use of SBS-based mats in food preservation and shelf-life extension^5^. SBS technology provides a suitable platform for integrating colorimetric indicators and facilitating their interaction with oxidation-related compounds. Therefore, SBS was chosen in this work as a practical and scalable approach for producing a flexible colorimetric sensor for monitoring oil oxidation.

Solution-blown spinning is receiving widespread attention in many fields and has become an effective and versatile method for manufacturing nanofiber materials with a controlled microstructure and shape^6,7^. One of the most important ingredients used daily in the food industry to add flavor and nutrition to food is edible vegetable oils. Edible oils and fats are a source of energy and essential fatty acids that are vital for our health, not just adding flavor to food^8,9^. Soybean oil is one of the edible vegetable oils, which is frequently used in the heating process and a main source of fried food flavor^10^.

The deterioration of oils is mainly due to oxidation. This oxidation reduces the shelf life of oils, renders them unusable, reduces the nutritional value of food, reduces sensory acceptance, and also leads to the formation of toxic compounds that cause health problems for consumers^11–13^. Given the importance of the quality of cooking oils and their impact on food quality, food industry leaders have recently been busy finding a way to determine oil validity. Developed an intelligent film for visually sensing lipid oxidation based on the colorimetric reaction^14^. Electrospinning nanofiber mats and developed a method to visually determine the oxidation of edible oils^15^. Liu et al. assessed the oxidation of edible oil using a chitin-based colorimetric sensor for aldehydes^16^.

A high-pressure optical mass spectrometry methodology was presented for the first time for the rapid detection of oxidative degradation in edible oils^17^. Several methods have been proposed to measure lipid oxidation. A methodology for assessing oil oxidation has been developed, based on wavelength absorption spectroscopy using Image-J intensity standardization. Furthermore, this method is not affected by any external factors or the method of use^18^. Oil oxidation is usually assessed and identified by changes in the oil’s physical, chemical, and sensory properties^19,20^. This includes traditional and modern analytical methods. On one hand, the determination of iodine (IV) value, acid value (AV), para-anisidine value (p-AV), peroxide value (POV), thiobarbituric acid (TBA), and total oxidation value (TOV). On the other hand, high-pressure liquid chromatography, Fourier transform infrared spectroscopy, gas chromatography olfactometry, and nuclear magnetic resonance spectroscopy^21–23^. All these methods and others take a long time, some are expensive, and require a laboratory environment, equipment, and laboratory personnel. Traditional methods are hindered by their need for laboratory procedures, chemical reagents, sample preparation, and specialized equipment, thus failing to provide rapid detection of spoiled oil. This has led to the use of colorimetric sensors for optical detection, as nano-mats offer a porous structure and a high surface area-to-volume ratio, facilitating the diffusion and interaction of volatile oxidation products with the sensing indicator. Previous studies have demonstrated the feasibility of using nanofiber-based colorimetric systems for the optical detection of oxidation in edible oils^5,15^.

Nowadays, visual detection with the naked eye is very promising, especially for ordinary consumers, after the rise of public concern. Production of biodegradable, smart food quality monitoring packaging based on pH-sensitive natural pigments has recently begun^24^. A flaxseed mucilage/sodium alginate composite film incorporating norbixin and WO₃ nanoparticles (Muc/Al/WO/NB) exhibited improved antioxidant activity, reduced oxygen and water vapor permeability, and enhanced physicochemical properties. The controlled release of norbixin further demonstrated the potential of this nanocomposite film as an active packaging material for food applications^25^. Nanostructured polyaniline (PANI)-coated fibers have been developed as resistive gas sensors for the rapid and simple detection of 1,1-dimethylhydrazine (DMH). The sensor exhibited good sensitivity and reproducibility, demonstrating the potential of conducting polymer-coated fibers for sensitive gas detection applications^26^. Cellulose nanofiber films incorporated with methylene blue and vitamin C were successfully developed as active colorimetric packaging for virgin olive oil. The Cel/MB/VC film reduced the increase in acidity and peroxide value while preserving phenolic compounds, and its visible white-to-blue color change showed a strong correlation with oil oxidation and storage time, demonstrating its potential for real-time monitoring and shelf-life estimation of edible oils^27^. A glucomannan-based biodegradable film incorporating turmeric and selenium was developed as a colorimetric ammonia sensor for food spoilage monitoring. The optimized film exhibited a rapid response (2 min), high sensitivity, good repeatability, and a clear visual color change toward ammonia. Its successful application in shrimp spoilage detection highlights the potential of bio-based composite films as smart packaging and visual freshness indicators^28^.

Congo red is recognized for its pH sensitivity, which permits obvious color variations in oxidative environments. To detect food safety, color sensors have recently been developed, whether in films, indicator strips, or labels^29^. This study addressed the production of color sensors from nanofiber mats using a solution-blown spinning technique, with varying spinning parameters during production.

**Fig. 1:**
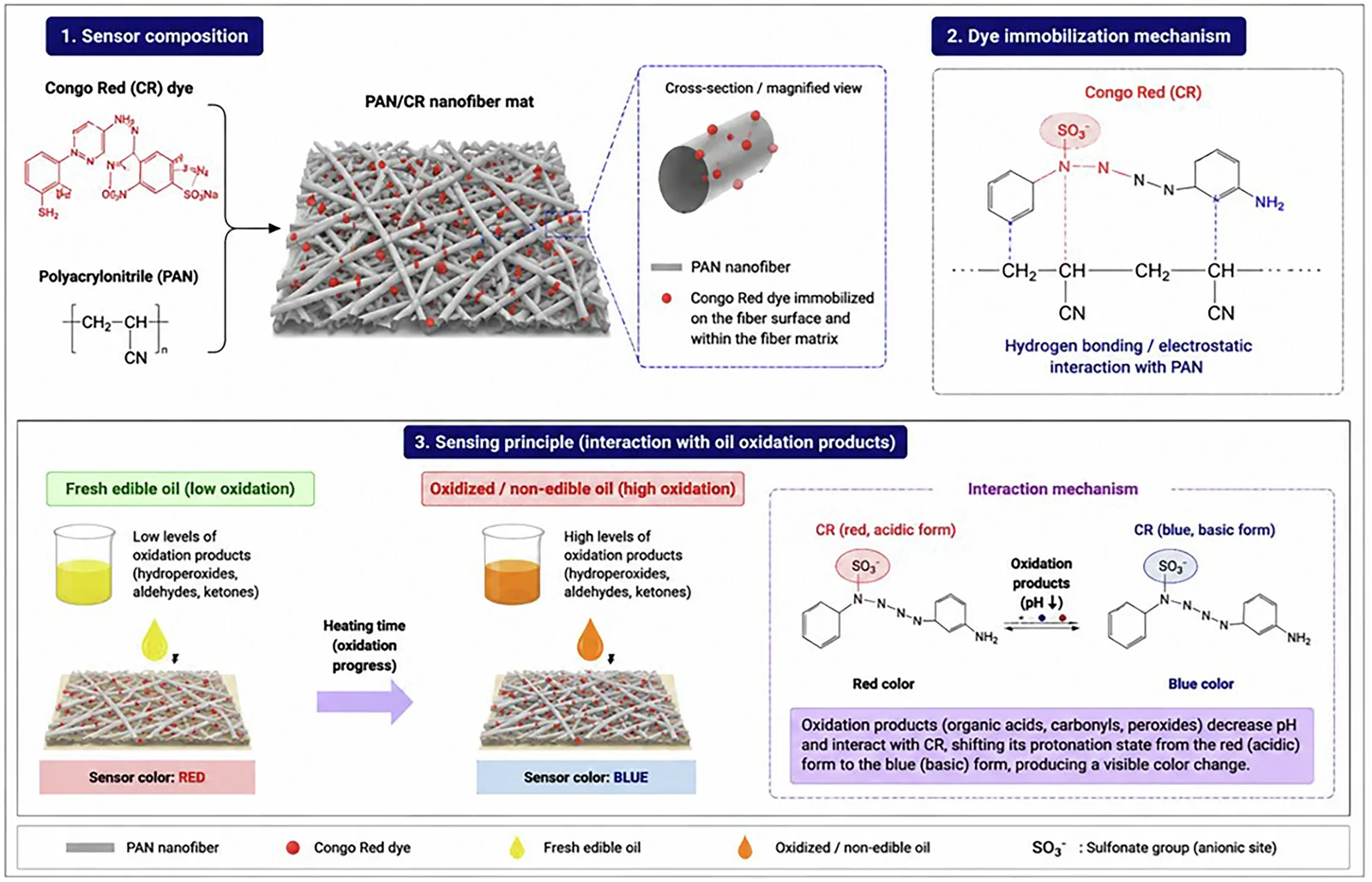
Schematic illustration of the PAN/CR nanofiber colorimetric sensor. Schematic representation of the PAN/Congo Red (CR) nanofiber colorimetric sensor, showing the incorporation of CR within the PAN nanofiber matrix and the proposed sensing mechanism for detecting soybean oil (SBO) oxidation.

The spinning solution also contains a sensitive dye (Congo red) to detect oil oxidation. The color of the sensors can change when the oil oxidizes, signaling to the consumer that the oil is unfit for use. The main objective is to evaluate the effect of different spinning parameters on the sensitivity and efficiency of nanofiber-based sensors in detecting oil oxidation and their suitability for use. This is achieved by establishing a relationship between the diameter of the fibers produced and the rate of visible color change. The Congo dye color changes from red to blue due to oils (Fig. 1). Oxidized samples of soybean oil (heated for 7 h at 180 °C) were assessed for their physicochemical features, including peroxide, para-anisidine, total oxidation, and acid values. The sensor changes color from pink to blue according to the oxidation degree, showing a simple visual sign for consumers, supported by the oxidation mechanism of the oil.

## Results

### Morphology and diameter characterization

**Fig. 2:**
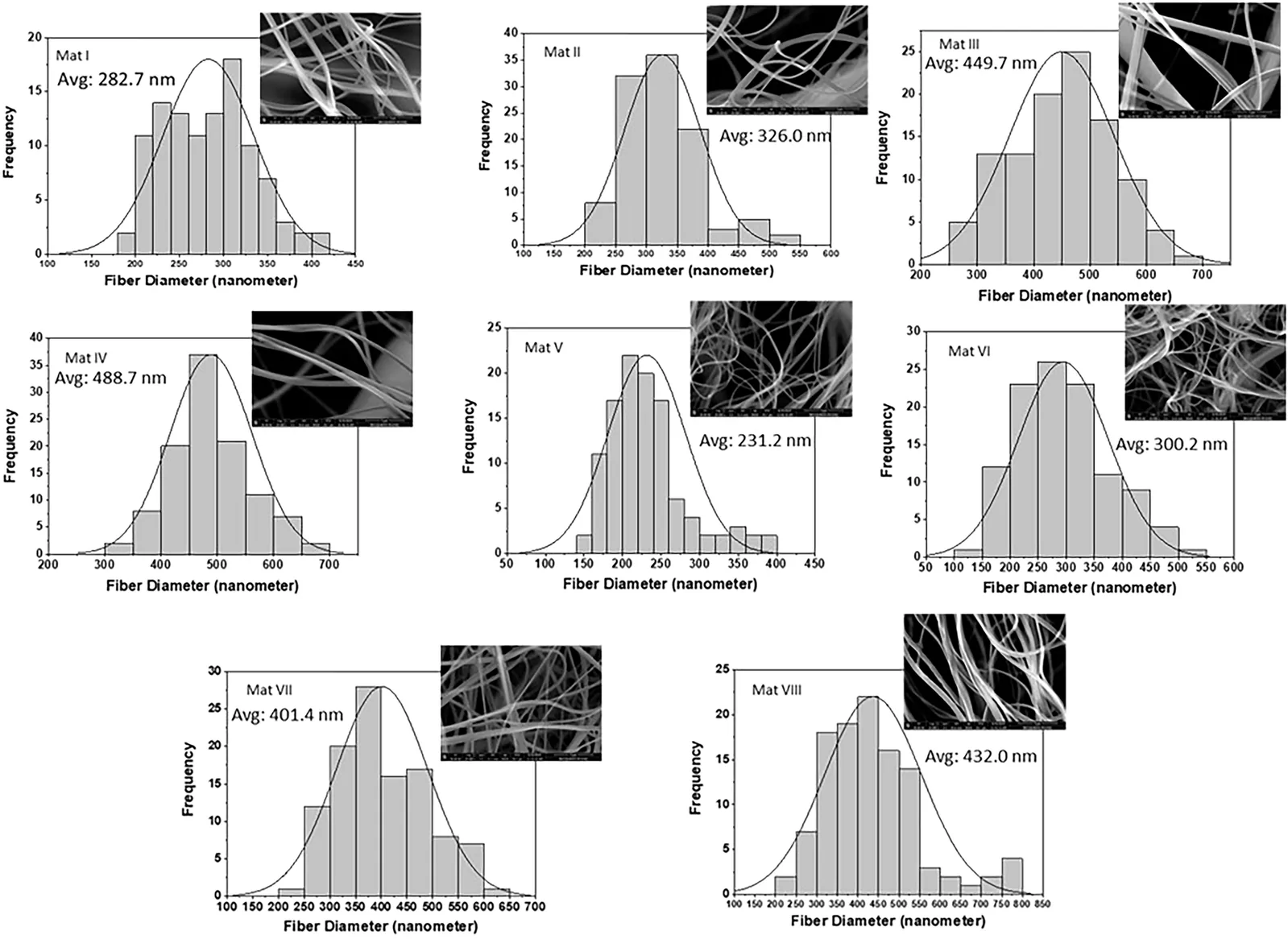
Morphology and fiber diameter distribution of PAN/CR nanofiber mats. SEM micrographs of nanofiber mats (MAT I–VIII) and their corresponding fiber diameter distributions. Images were acquired at a magnification of 20,000× using an accelerating voltage of 20.00 kV; the scale bar represents 5 μm.

The effect of spinning parameters on nanofiber diameter was investigated using different polymer concentrations, air pressures, and needle sizes. Scanning electron microscopy (SEM) was used to characterize the morphology and diameter of nanofibers (MAT I–VIII). Diameter distributions were determined from SEM images, and the mean nanofiber diameters are presented in Fig. 2 and Table 1.

**Table 1:** The composition of developed nanofiber mats (I-VIII)

No.	Needle gauge (G)	Con. of PAN (%)	Air pressure (Mpa)	Average Diameter with standard deviation (nanometers)
MAT I	18	12%	2	282.7 ± 51.7
MAT II	18	12%	0.5	326.0 ± 61.7
MAT III	18	15%	2	449.7 ± 92.7
MAT IV	18	15%	0.5	488.7 ± 72.5
MAT V	21	12%	2	231.2 ± 50.2
MAT VI	21	12%	0.5	300.2 ± 82.9
MAT VII	21	15%	2	401.4 ± 88.8
MAT VIII	21	15%	0.5	432.0 ± 118.4

### Effect of air pressure on nanofiber diameter

Two air pressures (0.5 and 2 MPa) were used for each polymer concentration and needle size. For samples with the same polymer concentration and needle size, increasing air pressure resulted in a decrease in nanofiber diameter (Fig. 2 and Table 1). Samples I and II showed mean diameters of 326.0 and 282.7 nm, respectively, corresponding to a 13.2% decrease with increasing air pressure. Similarly, the diameter decreased by 7.9% between samples III and IV, 22.9% between samples V and VI, and 7.0% between samples VII and VIII. Increasing air pressure from 0.5 to 2 MPa decreased the mean nanofiber diameter from 300.2 to 231.2 nm. The largest decrease was observed between samples V and VI.

### Effect of polymer concentration on nanofiber diameter

Increasing polymer concentration from 12 to 15% resulted in an increase in nanofiber diameter when air pressure and needle size were kept constant (Fig. 2 and Table 1). The diameter increased by 59.0% between samples I and III, 49.9% between samples II and IV, 73.6% between samples V and VII, and 44.0% between samples VI and VIII. The largest increase was observed between samples V and VII, where the mean diameter increased from 231.2 to 401.4 nm.

### Effect of needle size on nanofiber diameter

Needle size also affected the diameter of the produced nanofibers. Comparisons between samples I and V, II and VI, III and VII, and IV and VIII showed that the 18 G needle produced larger-diameter fibers than the 21 G needle under the corresponding spinning conditions. Among the fabricated mats, sample IV exhibited the largest mean diameter, whereas sample V exhibited the smallest mean diameter.

### FTIR analysis

**Fig. 3:**
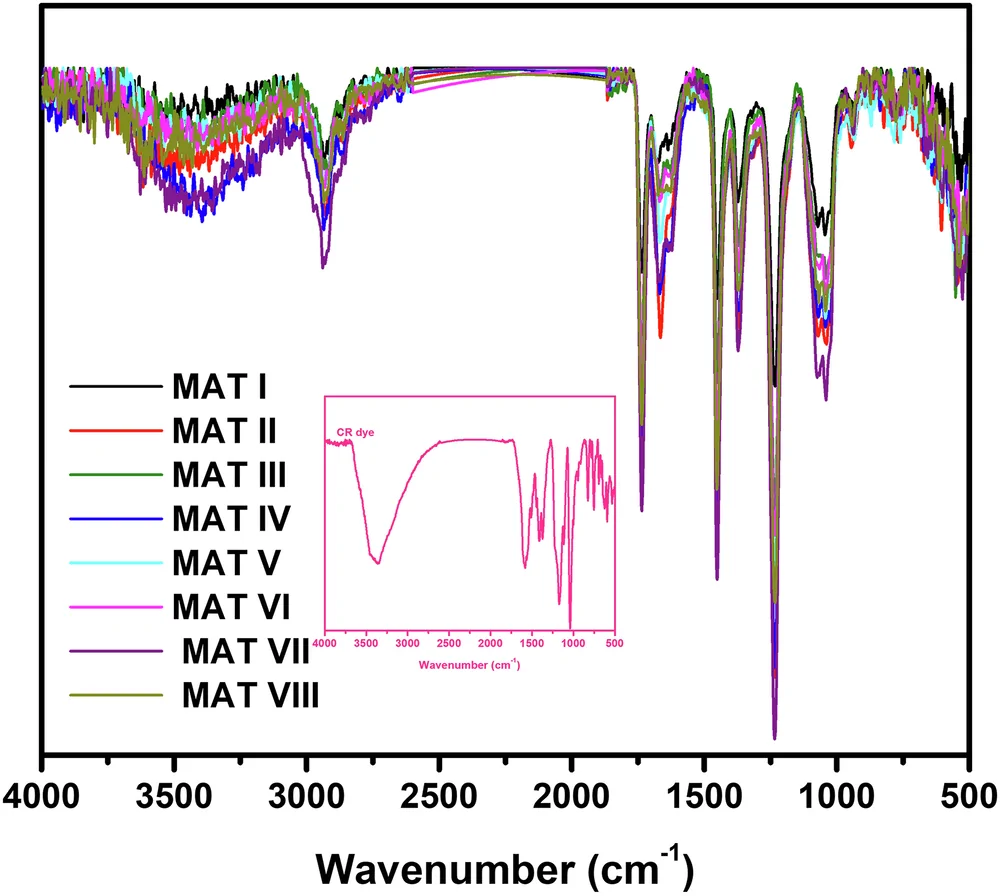
FTIR characterization of PAN/CR nanofiber mats fabricated under diff erent spinning conditions. FTIR spectra of PAN/CR nanofiber mats (MAT I–VIII) fabricated using different spinning parameters, including needle gauge (18 G or 21 G), PAN concentration (12% or 15%), and air pressure (0.5 or 2 MPa).

The FTIR spectra of the fabricated PAN/Congo red (CR) nanofiber mats (MAT I–VIII) are presented in Fig. 3. The spectra exhibited characteristic PAN absorption bands at approximately 2930–2945 cm^−1^, corresponding to C–H stretching vibrations, and at 1350–1450 cm^−1^, associated with CH_2_ and CH_3_ bending vibrations^30,31^. Following incorporation of CR into the PAN matrix, additional or intensified bands were observed at approximately 1130–1150, 1366–1375, and 1600–1620 cm^−1^, which were assigned to SO_3_^-^symmetric stretching, C–N stretching, and aromatic C *=* C stretching, respectively. Comparison of MAT I–VIII showed minor variations in peak positions within these ranges, while differences in peak intensity and band broadening were more evident. MAT IV and MAT VIII, prepared using 15% PAN and 0.5 MPa air pressure, exhibited relatively sharper and more distinct absorption bands. In contrast, MAT I, III, V, and VII, produced at 2 MPa air pressure, showed broader bands and minor variations in peak positions. The characteristic PAN bands were retained in all mats, with no pronounced spectral shifts indicating substantial alteration of the PAN chemical structure.

### X-ray diffraction

**Fig. 4:**
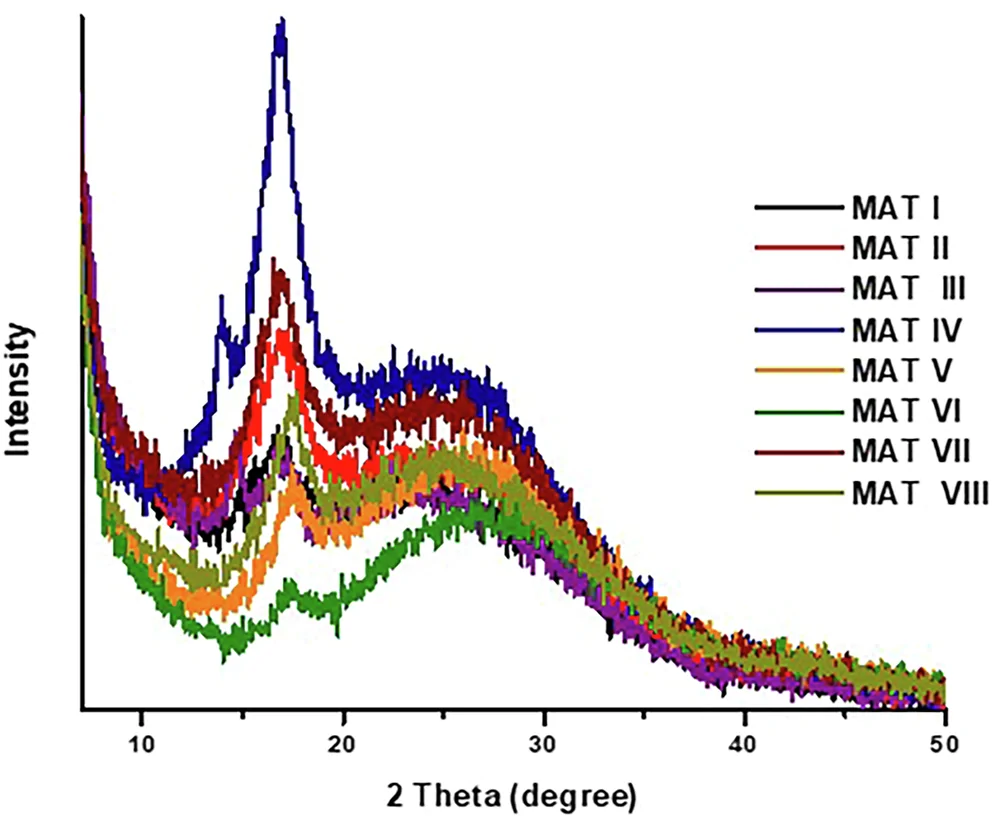
XRD patterns of PAN/CR nanofiber mats fabricated under diff erent spinning conditions. XRD patterns of PAN/CR nanofiber mats (MAT I–VIII) fabricated using different spinning parameters, including needle gauge (18 G or 21 G), PAN concentration (12% or 15%), and air pressure (0.5 or 2 MPa).

The X-ray diffraction patterns of the fabricated nanofiber mats (MAT I–VIII) are presented in Fig. 4. The mats exhibited similar overall peak positions, while differences were observed in diffraction intensity and relative crystallinity. MAT IV exhibited the highest relative crystallinity (46.8%), followed by MAT II and MAT VII (34.1%). The relative crystallinity values of MAT VIII, MAT I, MAT V, MAT III, and MAT VI were 25.9%, 21.1%, 14.4%, 13.9%, and 1.2%, respectively. Thus, MAT IV showed the highest degree of relative crystallinity, whereas MAT VI showed the lowest. Although MAT I and MAT III exhibited peak positions similar to those of MAT II and MAT VII, their diffraction intensities were lower. The differences in diffraction intensity and relative crystallinity indicate variations in the degree of molecular ordering among the fabricated nanofiber mats.

### Color analysis

The colorimetric characteristics of the solution-blown nanofiber mats produced under different spinning conditions are presented in Table 2. Changes in needle size, polymer concentration, and air pressure resulted in variations in the L^*^, a^*^, and b^*^ values among MAT I–VIII. MAT VII and MAT VIII exhibited the highest L* values, indicating greater lightness, whereas MAT I, MAT V, and MAT VI showed lower L^*^ values. The highest a* values, corresponding to stronger red coloration, were observed for MAT I, MAT II, and MAT IV, while MAT V and MAT VI exhibited lower a^*^ values. Differences were also observed in b^*^ values among the fabricated mats. Increasing polymer concentration resulted in higher b^*^ values under the corresponding spinning conditions. MAT III and MAT IV showed lower b^*^ values than MAT I and MAT II, while MAT VII and MAT VIII exhibited higher b^*^ values than MAT V and MAT VI. The results demonstrate that variations in spinning parameters produced measurable differences in the colorimetric properties of the PAN/CR nanofiber mats.

**Table 2:** Color analysis of developed nanofiber mats

MATS	L (light-dark)	a (red-green axis)	b (Yellow-Blue axis)
I	67.45 ± 0.07	20.9 ± 0.04	10.5 ± 0.03
II	70.05 ± 0.03	22.7 ± 0.01	11.2 ± 0.02
III	71.75 ± 0.03	18.4 ± 0.02	13.5 ± 0.01
IV	71.15 ± 0.14	20.4 ± 0.01	11.3 ± 0.04
V	67.5 ± 0.09	15.3 ± 0.02	10.1 ± 0.01
VI	68.6 ± 0.01	15.5 ± 0.01	8.95 ± 0.02
VII	78.5 ± 0.04	17.3 ± 0.03	14.1 ± 0.03
VIII	76.05 ± 0.02	18.7 ± 0.01	15.2 ± 0.03

### Soybean oil oxidative stability parameters

**Fig. 5:**
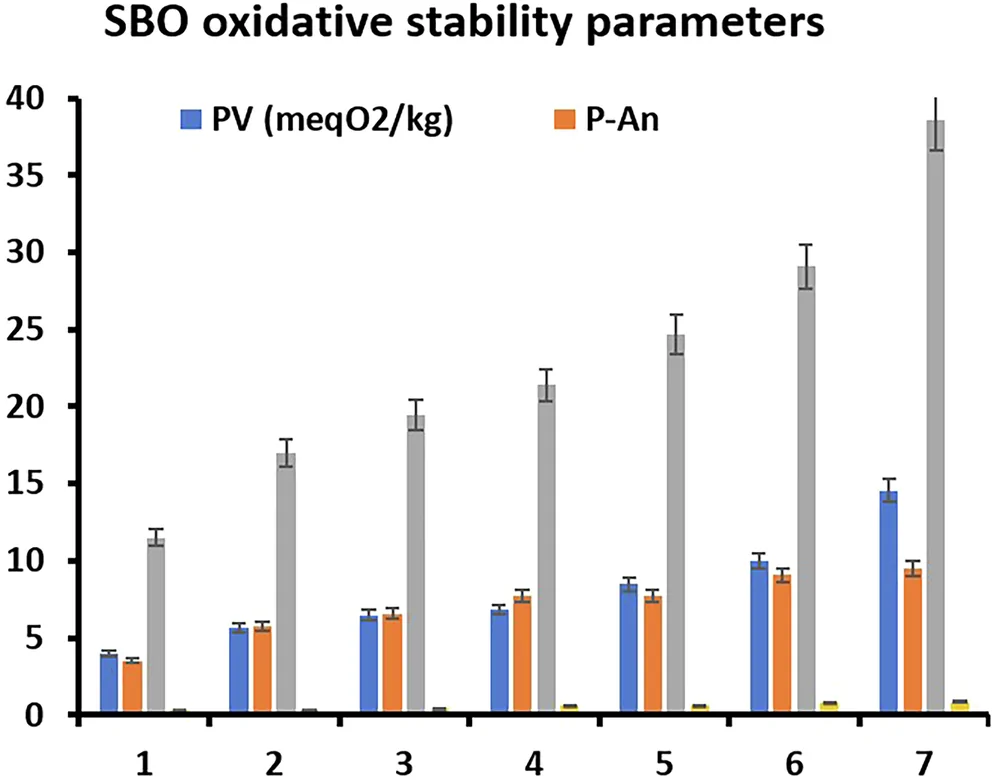
Changes in soybean oil oxidation parameters during thermal treatment. Effect of heating time (1–7 h) on the oxidative stability parameters of soybean oil (SBO), including peroxide value(PV), p-anisidine value (p-AV), and acid value (AV), for monitoring the progression of oil oxidation.

The oxidative stability of SBO during thermal treatment was evaluated by monitoring peroxide value (PV), p-anisidine value (P-An), total oxidation value (TOV), and acid value (AV) over 7 h of heating (Fig. 5). All measured parameters increased with increasing heating time. PV increased progressively during heating, reaching 14.55 meq O_2_/kg oil after 7 h. P-An also increased with heating time, from 3.52 at the beginning of treatment to 9.48 after 7 h. TOV showed a marked increase during heating, particularly after 5 h, and reached its highest value at 7 h. AV increased gradually from 0.28 to 0.84 mg KOH/g during the heating period. Prolonged thermal treatment resulted in progressive increases in the primary and secondary oxidation indicators, as well as the hydrolytic degradation indicator, demonstrating measurable deterioration of SBO during heating.

### Response and effectiveness of the colorimetric sensor

#### Effect of nanofiber diameter on sensor response time

**Fig. 6:**
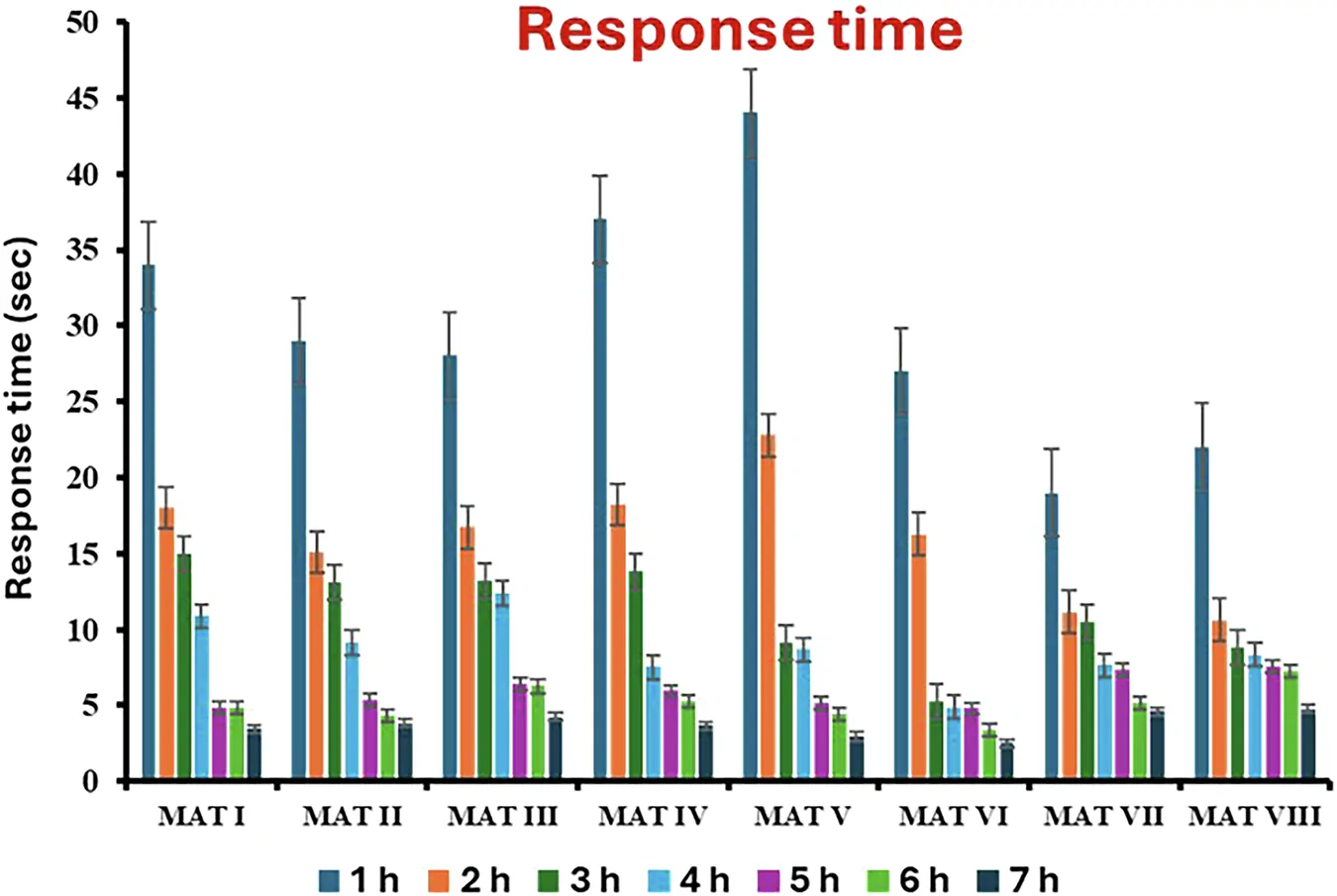
Response time of PAN/CR nanofiber mats to thermally oxidized soybean oil. Response time of different nanofiber mats (MAT I–VIII) used as colorimetric sensors for detecting oil oxidation after thermal treatment of SBO for 1–7 h. Data are presented as mean ± standard deviation (SD).

The response time of MAT I–VIII was evaluated after exposure to soybean oil heated for different durations (1–7 h) (Fig. 6). Response times varied according to both the heating duration and the nanofiber mat. For all mats, the response time was generally longer after 1 h of oil heating and decreased with increasing heating duration. A relationship was also observed between nanofiber diameter and response time. Mats with smaller nanofiber diameters generally exhibited shorter response times. MAT V and MAT VI, which were produced using a 21 G needle and 12% PAN, showed the shortest response times among the tested mats.

### Effect of oil oxidation parameters on sensor response time

Soybean oil was heated at 180 ± 2 °C for up to 7 h to generate different degrees of thermal oxidation. The response times of MAT I–VIII following exposure to the heated oil samples are shown in Fig. 6.

MAT I generally exhibited the longest response times and showed greater variation with increasing heating duration. MATs II and III showed intermediate response times. In contrast, MATs IV–VIII generally exhibited shorter response times with comparatively smaller variations across the heating periods. MATs VI–VIII provided relatively rapid and consistent responses at different oxidation levels. After 7 h of heating, PV, P-An, and AV reached 14.55 meq O_2_/kg oil, 9.48, and 0.84 mg KOH/g, respectively. Increasing heating duration was associated with shorter sensor response times, indicating an increased colorimetric response of the nanofiber mats to progressively oxidized oil.

### Effect of fiber diameter on sensor color changes

**Fig. 7:**
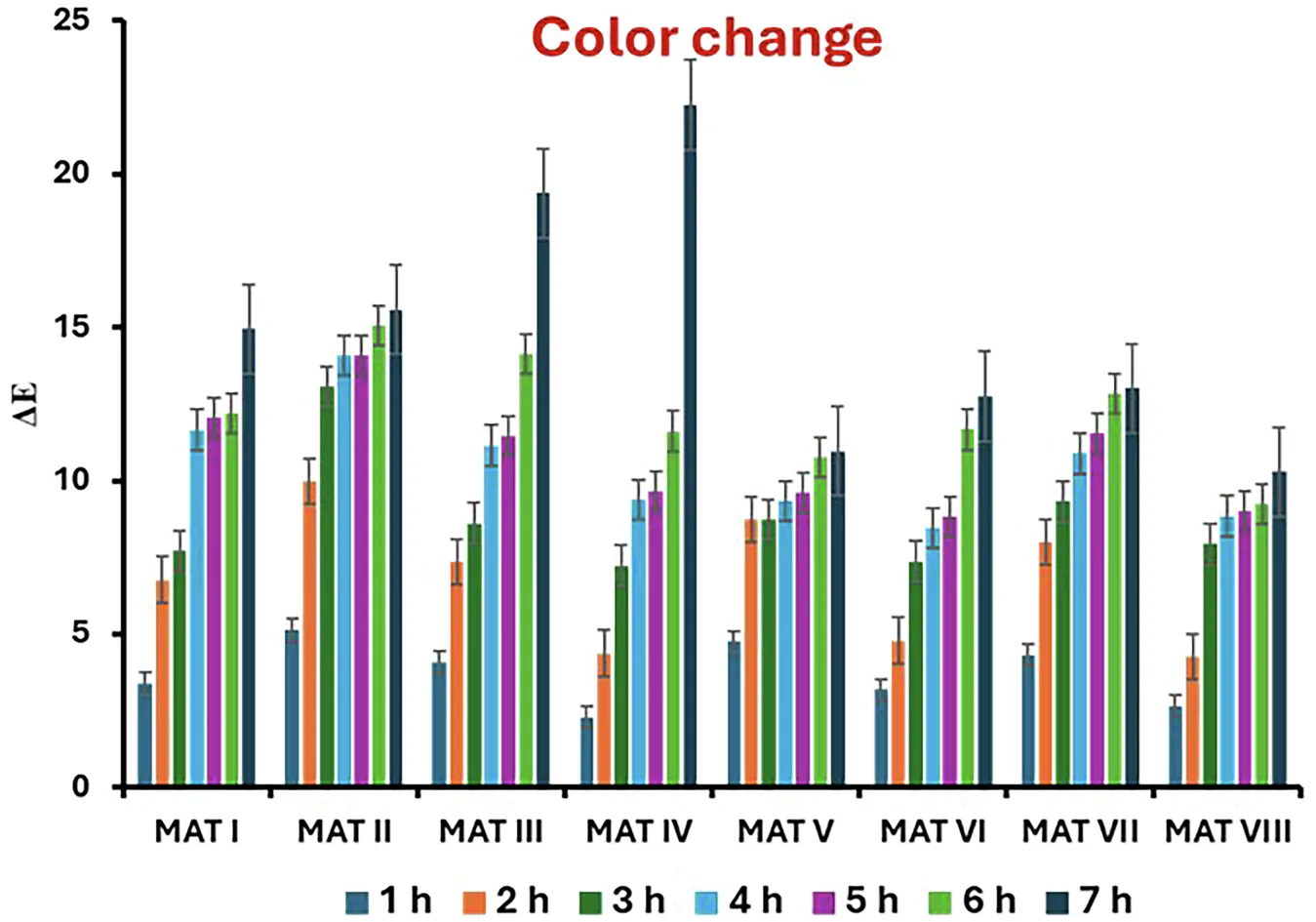
Colorimetric response of PAN/CR nanofiber mats to soybean oil oxidation. Color change response of different nanofiber mats (MAT I–VIII) used as visual indicators during soybean oil heating for 1–7 h, demonstrating the progressive colorimetric response associated with increasing oil oxidation.

The colorimetric response of the fabricated nanofiber mats to oxidized soybean oil was evaluated by monitoring changes in color difference (ΔE) after exposure to heated oil for up to 7 h (Fig. 7). All nanofiber mats exhibited a visible color change from red to blue following exposure to heated oil, although the magnitude of the change varied among the mats. MAT V and MAT VI showed the lowest ΔE values, 10.97 and 12.75, respectively. MAT I and MAT II exhibited intermediate ΔE values of 14.95 and 15.57, respectively. Higher ΔE values were observed for MAT III, MAT IV, MAT VII, and MAT VIII, with values of 19.38, 22.25, 23.02, and 20.29, respectively. The fabricated mats exhibited different degrees of color response following exposure to heated oil, with ΔE values ranging from 10.97 to 23.02.

## Discussion

The observed changes in nanofiber diameter can be attributed to the combined effects of air pressure, polymer concentration, and needle size. Increasing air pressure promotes greater stretching of the polymer jet during solution blowing, resulting in finer fibers. The reduction in fiber diameter observed with increasing air pressure is consistent with previous reports describing the influence of aerodynamic forces on fiber formation^32^.

Polymer concentration showed the opposite effect. The increase in fiber diameter at higher PAN concentrations can be attributed to increased polymer-chain entanglement and solution viscosity, which limits jet stretching and favors the formation of thicker fibers. Higher polymer concentration can also reduce solvent evaporation during fiber formation, contributing to the increased diameter^32,33^.

Needle size also influenced fiber formation. The larger 18 G needle produced thicker fibers than the smaller 21 G needle. This effect may be related to differences in the initial solution flow and the interaction between the polymer solution and the surrounding high-velocity air stream. Needle geometry and positioning can affect jet stability and fiber uniformity in solution-blowing systems^34^.

The results demonstrate that the spinning parameters can be effectively controlled to tailor nanofiber diameter. Because fiber diameter can influence the surface area and interaction of the Congo red-containing nanofiber mat with oxidation-related compounds, controlling these parameters is important for optimizing the colorimetric sensing performance.

The FTIR results support the incorporation of Congo red within the PAN nanofibrous matrix. The appearance or intensification of CR-associated bands, together with the retention of the characteristic PAN bands, indicates that CR was incorporated without substantial chemical degradation or major alteration of the PAN structure. The differences in peak intensity and band broadening among the mats are more likely related to differences in nanofiber morphology, polymer-chain organization, and CR distribution resulting from the different solution-blowing conditions.

The sharper spectral features observed for mats produced at higher polymer concentration and lower air pressure may be associated with greater polymer-chain entanglement and a more organized nanofibrous structure. In contrast, the broader features observed at higher air pressure may reflect the effects of increased jet stretching and rapid solvent evaporation on the physical organization of the fibers^35^. Increasing PAN concentration also increases solution viscosity and polymer-chain entanglement, which can favor the formation of thicker and more uniform fibers and may facilitate a more homogeneous distribution of CR within the matrix. The differences between mats produced using 18 G and 21 G needles further indicate that needle geometry and the resulting fiber morphology can influence the physical organization and distribution of CR. These observations are consistent with the SEM results, which demonstrated that spinning parameters affected nanofiber diameter and morphology.

The FTIR findings also support the proposed physical retention of CR within the PAN nanofibrous network. CR may be retained primarily through physical entrapment within the interconnected fiber structure, while the spectral changes may suggest possible non-covalent interactions between CR and PAN, including hydrogen-bonding or dipole-related interactions. However, FTIR alone cannot establish the strength or specific nature of these interactions or quantitatively confirm dye retention. In particular, a quantitative dye-leaching study was not performed; therefore, CR release during prolonged contact with oxidized oil cannot be conclusively determined. Future studies should evaluate CR release under relevant application conditions. The fabricated mats were stored under dry conditions and protected from direct light before use. Nevertheless, long-term storage stability and sensor shelf life were not systematically evaluated. Future studies should therefore assess changes in morphology, FTIR characteristics, colorimetric response, and CR retention under controlled temperature and relative humidity conditions.

The differences in relative crystallinity among the fabricated mats indicate that the solution-blowing parameters influenced the molecular organization of the PAN nanofibers. The relatively high crystallinity observed for MAT IV, MAT II, and MAT VII may be associated with the polymer concentration and specific processing conditions, which can promote polymer-chain entanglement and molecular ordering. During solution blowing, the applied aerodynamic forces can stretch the polymer jet and influence polymer-chain alignment as the solvent evaporates. Such chain alignment may contribute to the development of more ordered regions within the nanofibers^36^. However, the relationship between air pressure and crystallinity is not necessarily linear because crystallinity is also affected by polymer concentration, needle size, jet stretching, solvent evaporation, and the resulting fiber morphology.

The very low relative crystallinity observed for MAT VI suggests a comparatively disordered molecular structure under its specific combination of spinning parameters. These differences in molecular organization may contribute to variations in the physical properties of the nanofiber mats and could potentially influence their sensing performance by affecting fiber morphology, surface characteristics, and dye distribution. The observed differences in L^*^, a^*^, and b^*^ values can be attributed to variations in nanofiber diameter, fiber packing, surface morphology, and Congo red distribution resulting from the different spinning conditions. Mats VII and VIII showed higher lightness, which may reflect a more uniform fiber structure and greater surface reflectance. In contrast, the lower L^*^ values observed for some mats may be associated with denser fiber structures, aggregation, or less uniform fiber formation. The stronger red coloration observed for MAT I, MAT II, and MAT IV may be related to differences in fiber diameter and the distribution of Congo red within the nanofibrous matrix. The lower a^*^ values observed for MAT V and MAT VI may reflect differences in dye distribution associated with their finer fiber structures and greater surface area.

Polymer concentration also influenced the b^*^ values. Increasing PAN concentration increases solution viscosity and polymer-chain entanglement, which can affect fiber formation, surface uniformity, and light scattering. These changes may explain the higher b* values observed for the mats produced at higher polymer concentration. Air pressure and needle size can additionally affect jet stability and fiber diameter, thereby influencing the optical properties of the resulting mats. However, the colorimetric differences should primarily be interpreted as changes in the physical and optical characteristics of the mats rather than direct evidence of changes in the chemical state of Congo red. The combined effects of fiber morphology and dye distribution are therefore important when selecting the optimal spinning conditions for subsequent colorimetric detection of oil oxidation.

The progressive increase in PV indicates the accumulation of hydroperoxides as primary oxidation products during thermal treatment. The increase in P-An alongside PV suggests that the hydroperoxides formed during the early stages of oxidation subsequently decomposed into aldehydes and other secondary oxidation products as heating continued. This progression is consistent with the expected development of lipid oxidation during prolonged thermal exposure.

The marked increase in TOV, particularly after 5 h of heating, reflects the simultaneous accumulation of primary and secondary oxidation products and therefore provides an overall indication of the deterioration of the oil. The results are consistent with previous studies showing that prolonged heating accelerates oxidative deterioration and can adversely affect the nutritional and sensory quality of edible oils^37–39^.

The gradual increase in AV indicates that thermal treatment also promoted hydrolytic degradation, leading to the release of free fatty acids. Although the change in AV was less pronounced than those observed for PV and P-An, it provides additional evidence that thermal treatment affected both oxidative and hydrolytic stability. Taken together, the simultaneous changes in PV, P-An, TOV, and AV demonstrate progressive deterioration of SBO during heating. These changes provide an appropriate reference for evaluating the response of the PAN/CR nanofiber mats to different stages of oil oxidation.

The reduction in response time with increasing heating duration can be attributed to the greater accumulation of oxidation products in the heated oil. As thermal oxidation progresses, primary oxidation products undergo further decomposition, generating secondary compounds such as aldehydes and other volatile oxidation products. The higher concentration of these oxidation-related compounds may facilitate their interaction with the Congo red-containing nanofiber matrix and consequently accelerate the visible color response.

Nanofiber diameter also appears to influence sensor response. Finer nanofibers provide a higher specific surface area and a shorter diffusion pathway, which may facilitate interaction between the sensor surface and oxidation-related compounds. This may explain the relatively rapid response observed for the finer mats, particularly MAT V and MAT VI. However, response time should not be considered independently of other performance characteristics, as fiber morphology, dye distribution, and matrix structure may also contribute to the observed response.

The progressive changes in PV, P-An, and AV provide an analytical reference for interpreting the sensor response. The increase in PV reflects the formation of primary oxidation products, whereas the increase in P-An indicates accumulation of secondary oxidation products. The simultaneous increase in AV indicates some contribution from hydrolytic degradation. Therefore, the shorter response times observed at longer heating durations may reflect the greater abundance of oxidation-related compounds available to interact with the sensor.

Among the fabricated mats, MATs VI–VIII showed a favorable combination of relatively rapid and consistent responses. In particular, MAT VIII was selected as the most favorable formulation when sensitivity and response stability were considered together. These findings demonstrate the potential of PAN/CR nanofiber mats for rapid indirect-contact visual monitoring of cooking-oil oxidation. However, the sensor response should be further validated against established oxidation markers and under a wider range of real food-use conditions before practical application.

The observed color transition from red to blue confirms the visual response of the Congo red-containing nanofiber mats to oxidized oil. Congo red has previously been reported to undergo a color change upon interaction with oxidized oil, supporting its potential application as a colorimetric indicator of oil oxidation^16^. Similar colorimetric behavior has also been reported for PAN/Congo red nanofiber mats^29^.

The differences in ΔE among the mats indicate that the magnitude of the color response was influenced by the characteristics of the nanofibrous matrix. Fiber diameter may contribute to this behavior by affecting surface area, fiber packing, and the accessibility and distribution of Congo red within the mat. However, because fiber diameter was simultaneously influenced by needle size, polymer concentration, and air pressure, the differences in ΔE should be interpreted as the combined effect of the spinning conditions rather than fiber diameter alone.

MAT V and MAT VI exhibited the lowest ΔE values despite showing relatively rapid response times. This indicates that response time and magnitude of color change represent different sensor-performance characteristics. A rapid response does not necessarily correspond to the largest ΔE value. Therefore, both response time and color-change magnitude should be considered when selecting the most suitable nanofiber formulation.

The higher ΔE values observed for MAT III, MAT IV, MAT VII, and MAT VIII suggest a stronger visible color response under the tested conditions. The differences may be associated with variations in fiber morphology and Congo red distribution resulting from the corresponding spinning parameters. Overall, the findings demonstrate that controlling the nanofiber fabrication conditions can modulate the visual response of the PAN/CR sensor and provide a basis for optimizing its performance for indirect detection of cooking-oil oxidation.

In conclusion, the developed nanofiber mats were successfully applied for the rapid detection of soybean oil oxidation. FTIR analysis supported the successful incorporation of Congo Red into the PAN nanofibers. The study demonstrated that varying the solution-blowing parameters significantly affected the sensitivity and response time of the nanofiber mats for detecting cooking oil oxidation. The response times varied depending on the nanofiber mat and oxidation period, ranging from approximately 3 to 44 s. Variations in the spinning parameters also affected the diameter of the produced nanofibers, with finer fibers generally showing higher sensitivity. Overall, the developed PAN/Congo Red nanofiber mats provide a rapid and non-contact visual sensing approach for monitoring cooking oil oxidation and may have potential applications in smart food-packaging and food-quality monitoring systems.

## Methods

### Materials

In this work, textile industry waste was used as a source for obtaining polyacrylonitrile polymer (PAN). Hydroxylamine hydrochlorides were purchased from Sigma-Aldrich (St. Louis, MO, USA). Dimethylformamide (DMF) was purchased from Fine Chem Ltd., Mumbai (India). Congo red dye (CR) was obtained from the local market (Giza, Egypt). Refined, bleached, and deodorized soybean with antioxidant addition was obtained from the local market. All other reagents and solvents were purchased from different suppliers.

### Fabrication of PAN/ CR nanofiber mats

To prepare nano mats for this study, the solution blowing spinning technique available at the Textile Alliance at the National Research Centre was used. The spinning machine was set as follows to study the effect of different spinning parameters on the sensitivity of the mats to oil oxidation: the flow rate was 15 mL/h, and the air pressure was 0.5 and 2 bar. The distance between the spinning nozzle and the nanofiber collector was fixed at 30 cm.

**Fig. 8:**
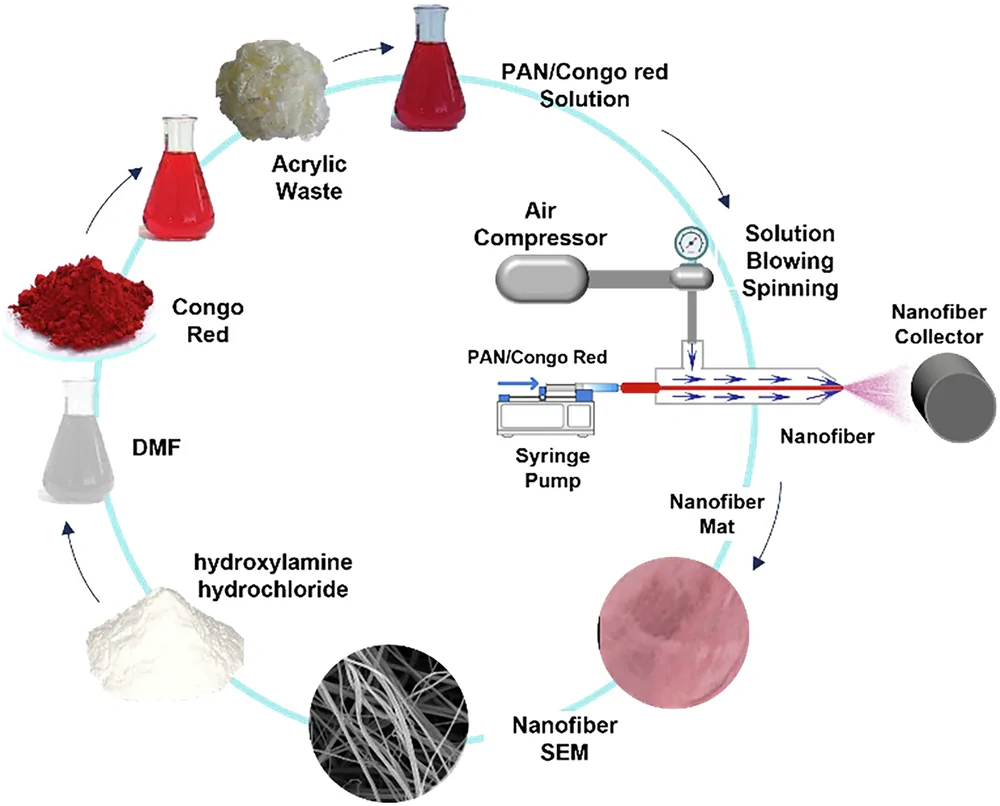
Fabrication of PAN/CR nanofiber mats by solution-blowing spinning. Schematic illustration of the fabrication process of PAN/CR nanofiber mats using the solution-blowing spinning technique, showing the main components and the formation and collection of the nanofibers.

To prepare PAN/CR nanofiber mats, 0.25 g of hydroxylamine hydrochloride was dissolved in 30 mL of DMF with stirring, and then 0.015 g of CR dye was added and mixed well. The amounts of hydroxylamine hydrochloride (0.25 g) and Congo Red (0.015 g) were selected based on preliminary formulation trials to obtain a stable spinning solution, good incorporation of the dye into the PAN matrix, and a distinct colorimetric response toward oxidized oil. These concentrations were therefore used consistently for all nanofiber mats, while the effects of PAN concentration, needle gauge, and air pressure were systematically investigated. Finally, PAN polymer was added at two different concentrations, 12 and 15%. Table 1 illustrates the prepared samples with different spinning parameters. Fig. 8.shows the steps of the fabrication of PAN/ CR nanofiber mats.

### Characterization of developed nanofiber mats

#### Morphology and diameter characterization

During the study, the samples were examined microscopically using a scanning electron microscope (SEM) to study the effect of different spinning parameters on the diameter of the produced nanofibers and the extent to which this is reflected in the sensitivity of the nanofiber mats to oil oxidation. Samples were prepared for SEM by coating them with gold, and then the samples were securely fixed using standard double-sided tape to the 0.5-inch diameter microscope nozzle. A JEOL JXA-480 SEM (JEOL, Tokyo, Japan) at the National Research Centre, Textile Science and Services Unit, Giza, Egypt, was used for the examination. The microscope operated at 20 kV and had a magnification of 8000x.

### Infrared spectroscopy analysis

To determine the change in the chemical functional groups during the nanofiber mats preparation and confirm the presence of CR dye, a Fourier Transform Infrared Analysis (FTIR) was performed. The test was conducted at the National Research Centre using a Bruker Vertex 80-volt spectrometer with a resolution of 4000–400 cm^−1^.

### X-Ray diffraction

XRD of the developed nanofiber mats was investigated using a Panalytical–Empyrean, Netherlands, equipped with CuKα Radiation (λ = 1.5406 Å). The samples were determined at a scanning degree of 0.1° in the 2θ range from 5° to 80° and a step time of 1 s. The relative crystallinity (X_c_) of the nanofiber mats was calculated from the XRD patterns using the following area-based equation:

1 $${\rm{X}}_{\rm{c}}( \% )=\frac{{\mathrm{A}}_{\rm{c}}}{\left({\mathrm{A}}_{{\rm{c}}}+{\mathrm{A}}_{{\rm{a}}}\right)}{\rm{x}}100$$

where *A*_c_ is the integrated area of the crystalline region, and *A*_a_ is the integrated area of the amorphous region.

### Colorimetric analysis

The L* (light-dark), a* (red-green), and b* (yellow-blue) values of the samples were measured using a colorimeter (Minolta CR-10, Japan). The equipment was calibrated using a white tile before doing the analysis.

### Thermal oxidation of soybean oil

500 mL of soybean oil was poured into a shallow stainless-steel pan with a diameter of 20 to 30 cm, followed by heating the oil at 180 °C ± 2 °C for 7 h. 50 mL of the heated oil was taken into a glass bottle after each hour, and rapidly kept at −20 °C for further examination.

### Determination of the oxidative stability of oil samples

The peroxide value, para-anisidine value, acid value, and volatile compounds were measured during the primary and secondary stages of oxidation to determine the oxidative stability of oils.

### Peroxide value determination

The peroxide value (PV) is determined to assess the storage properties of oils and fats. This value represents the total number of primary oxidation products existing in edible oils. Also, PV is measured to identify the peroxides and hydroperoxide concentration in the primary steps of lipid oxidation. The peroxide value was determined as follows: 1 g of the oil sample was dissolved in 25 mL of acetic acid: chloroform (3:2, v/v). After that, 1 mL of saturated potassium iodide solution was added, and the mixture was rapidly mixed by hand. The mixture was kept to stand for 3 min in a dark place, and then 40 mL of distilled water was added. 1 mL of starch (1%) was used as an indicator and added to the mixture. Sodium thiosulfate was used to titrate the mixture until it turned from dark violet to white. The peroxide value was calculated using the following equation:

2 $$\mathrm{PV}=\frac{\left({V}_{{\rm{s}}}-{V}_{{\rm{b}}}\right){\rm{X}}\;1000\;{\rm{X}}\;N}{W}$$

Where *V*_s_ and *V*_b_ are the sodium thiosulfate volumes taken by the sample and blank, respectively (mL). *N* is the normality of sodium thiosulfate (0.01 N); *W* is the oil sample weight in grams.

### Para-anisidine value

The p-anisidine value (p-AV) examination was used to assess the secondary oxidation products in the oil samples, following the AOCS method (AOCS, 1998). About 0.1 g of the oil sample was weighed and mixed with hexane (5 mL). The sample absorbance was measured against a hexane blank (A1) at 350 nm. Another oil sample (0.1 g) was mixed with 5 mL of hexane and added to 1 mL of a p-anisidine solution (2.5 g/L). The mixture was left in the dark for 10 min before absorbance measurement against a blank reagent (A2) at 350 nm. Thep-AV was then calculated using Eq. (3):

3 $${\boldsymbol{P}}-{\boldsymbol{AV}}={\bf{5}}{\boldsymbol{X}}\frac{{\bf{1.2}}\,{\boldsymbol{X}}\left({\boldsymbol{A}}{\bf{2}}-{\boldsymbol{A}}{\bf{1}}\right)}{{\boldsymbol{g}}}{\boldsymbol{oil}}$$

### Total oxidation value

The total oxidation value (TOV) is measured as the total oxidation profile of an oil sample. TOV is calculated from the PV and p-AV values, showing the variations in PV and p-AV values with heating time. TOV was determined using the following Eq. (4):

4 $${TOV}=\left(2\,X\,{PV}\right)+P-{AV}$$

### Acid value determination

The method was done through the AOAC (2005) method as follows: 0.10 N potassium hydroxide (KOH)solution was used to neutralize a mixture of diethyl ether and absolute ethanol1:1 (v/v), using phenolphthalein (1%) as an indicator. A pink color that continued for about 15 s showed the endpoint. For the test, 1 g of the oil sample was dissolved in of the neutralized ethanol–diethyl ether mixture 50 mL and titrated with the KOH solution under continual shaking. The free fatty acid (FFA) content, stated as an oleic acid percentage, was determined using the following Eq. (5):

5 $${\rm{FFA}} \% \;{\rm{as\; oleic\; acid}}=\frac{{\rm{N\; x\; S\; x}}\;28.2}{{\rm{W}}}$$

Where *N* represents the KOH normality, *S* means mL of 0.10 N KOH, and *W* equals the weight of the oil sample in g.

### Response and effectiveness of PAN nanofiber mats to oxidized oil samples

The nanofiber mats with aluminum foil were cut into a circular shape and put into the vial cap without coming into contact with the oil samples. At the end of each heating hour, the hot, oxidized soybean oil sample (2 g) was taken from the pan and placed inside a sealed 20-mL vial to record the response time. After 3 min, the color change for all samples was measured to unify the time of exposure.

### Colorimetric analysis

The color variance (ΔE) between the fiber mats before and after exposure to oxidized soybean oil samples was measured using the colorimeter (CR-10, Konica Minolta Sensing, Inc., Sakai, Osaka, Japan). The color change for all samples was measured after 3 min to unify the exposure time. The nanofiber mats were put into an empty 20-mL vial for the control sample. After calculating the color parameters L^*^ (lightness), a^*^ (red-green), and b^*^ (yellow-blue), the color difference (ΔE) was calculated using the following formula:

6 $${\mathbf{\Delta }}{\bf{E}}=\sqrt{{{({\mathbf{\Delta }}{\bf{L}}}^{* })}^{{\bf{2}}}+{{({\mathbf{\Delta }}{\bf{a}}}^{* })}^{{\bf{2}}}+{{({\mathbf{\Delta }}{\bf{b}}}^{* })}^{{\bf{2}}}}$$

Where ΔL^*^ = L^*^ − L^0*^, Δa^*^ = a^*^ − a^0*^, Δb^*^ = b^*^ − b^0*^. The color values of the nanofiber mats before the oxidized oil exposure are L^0^, a^0^, and b^0^, while the color values after the oxidized oil exposure are L^*^, a^*^, and b^*^.

### Statistical analysis

The significant differences between the samples due to spinning parameters were assessed using Duncan’s new multiple-range test at *p* < 0.05 using Statistica 6 software (StatSoft Inc., Tulsa, OK, USA) and one-way analysis of variance (ANOVA). Data were presented as the mean of three replicates. Error bars are shown in the corresponding figures.

## Data Availability

The datasets used and/or analysed during the current study available from the corresponding author on reasonable request
